# Differential Effects of Serum Lipoprotein-Associated Phospholipase A2 on Periventricular and Deep Subcortical White Matter Hyperintensity in Brain

**DOI:** 10.3389/fneur.2021.605372

**Published:** 2021-03-08

**Authors:** Junying Jiang, Yuanyuan Gao, Rui Zhang, Lin Wang, Xiaoyuan Zhao, Qi Dai, Wei Zhang, Xiujian Xu, Xuemei Chen

**Affiliations:** ^1^Department of Neurology, The Affiliated Jiangning Hospital With Nanjing Medical University, Nanjing, China; ^2^Department of General Practice, The Affiliated Jiangning Hospital With Nanjing Medical University, Nanjing, China

**Keywords:** lipoprotein-associated phospholipase A2, periventricular white matter hyperintensity, deep subcortical white matter hyperintensity, Fazekas score, pathogenesis of PVWMH

## Abstract

**Background and Purpose:** Serum level of lipoprotein-associated phospholipase A2 (Lp-PLA2) was associated with white matter hyperintensity (WMH). There were differences in the anatomical structure and pathophysiological mechanism between periventricular WMH (PVWMH) and deep subcortical WMH (DSWMH). In this study, we aimed to investigate the effects of serum Lp-PLA2 on the PVWMH and DSWMH.

**Methods:** In total, 711 Chinese adults aged ≥45 years with cranial magnetic resonance imaging (MRI) were recruited in this cross-sectional study, who had received physical examinations in the Department of Neurology, the Affiliated Jiangning Hospital of Nanjing Medical University due to dizziness and headaches between January 2016 and July 2019. Enzyme linked immunosorbent assay (ELISA) was utilized to determine the serum Lp-PLA2. Fazekas scale was used to measure the severity of PVWMH (grade 0–3) and DSWMH (grade 0–3) on MRI scans. Ordinal regression analysis was carried out to investigate the relationship between serum Lp-PLA2 and PVWMH or DSWMH.

**Results:** Finally, 567 cases were included in this study. The average level of serum Lp-PLA2 was 213.35±59.34 ng/ml. There were statistical differences in the age, hypertension, diabetes mellitus, atrial fibrillation, lacunar infarction, Lp-PLA2 grade, creatinine, Hcy, and H-CRP (*P* < 0.05) in PVWMH groups. Ordinal regression analysis indicated that there was a lower risk of PVWMH in the patients with normal and moderately elevated serum Lp-PLA2 compared with those with significantly elevated serum Lp-PLA2 after adjusting age, hypertension, diabetes mellitus, atrial fibrillation, lacunar infarction, Cr, Hcy, and H-CRP. In addition, PVWMH was correlated to advanced age, hypertension, diabetes mellitus, and lacunar infarction. After adjusting for confounding factors, DSWMH was correlated to advanced age and lacunar infarction. There was no correlation between serum Lp-PLA2 and DSWMH.

**Conclusions:** Serum Lp-PLA2 was closely associated with the pathogenesis of PVWMH rather than DSWMH. There might be different pathological mechanisms between PVWMH and DSWMH.

## Introduction

White matter hyperintensity (WMH), one the most common types of cerebral small vessel disease (CSVD) (1, 2), is mainly diagnosed based on the presence of hyperintensity on T2-weighted sequences, isointensity or hypointensity on T1-weighted signals unlike cerebrospinal fluid. It depends on the sequence parameters and the severity of pathological changes (3). To date, WMH shows a high prevalence in the aged population (4–6), which affects stroke outcome (7–9), cognitive impairment (10, 11), and several neurological symptoms (12, 13). Therefore, it is necessary to screen the risk factors associated with the pathogenesis of WMH.

Previous studies confirmed a relationship between inflammation and pathogenesis of WMH (14–16). Recently, a prospective study involving 15,792 adults aged between 45 and 65 years, midlife inflammation may lead to deterioration of WMH among the aged population (17). As an enzyme secreted by the circulating macrophages, lipoprotein-associated phospholipase A2 (Lp-PLA2) participated in the low density lipoprotein metabolism, contributed to the onset of atherosclerosis, and mediated the inflammation (18, 19). In a previous study, homocysteine (Hcy) concentration was associated with the increased burden of WMH (20). However, studies on the relationship between Lp-PLA2 and WMH are relatively few. According to the lesion sites, WMH could be divided into periventricular WMH (PVWMH) and deep subcortical WMH (DSWMH) (20). Generally, these two types of WMH are usually simultaneously developed and progressed. Nevertheless, increasing studies indicated that PVWMH and DSWMH showed various anatomical and histopathological changes, which demonstrated that there might be differences in their pathogenesis (20, 21). To date, no studies focused on the effects of serum Lp-PLA2 levels on the variations between PVWMH and DSWMH. In this study, we aimed to determine the impacts of serum Lp-PLA2 level on the onset of PVWMH and DSWMH.

## Methods

### Subjects

**Figure 1:**
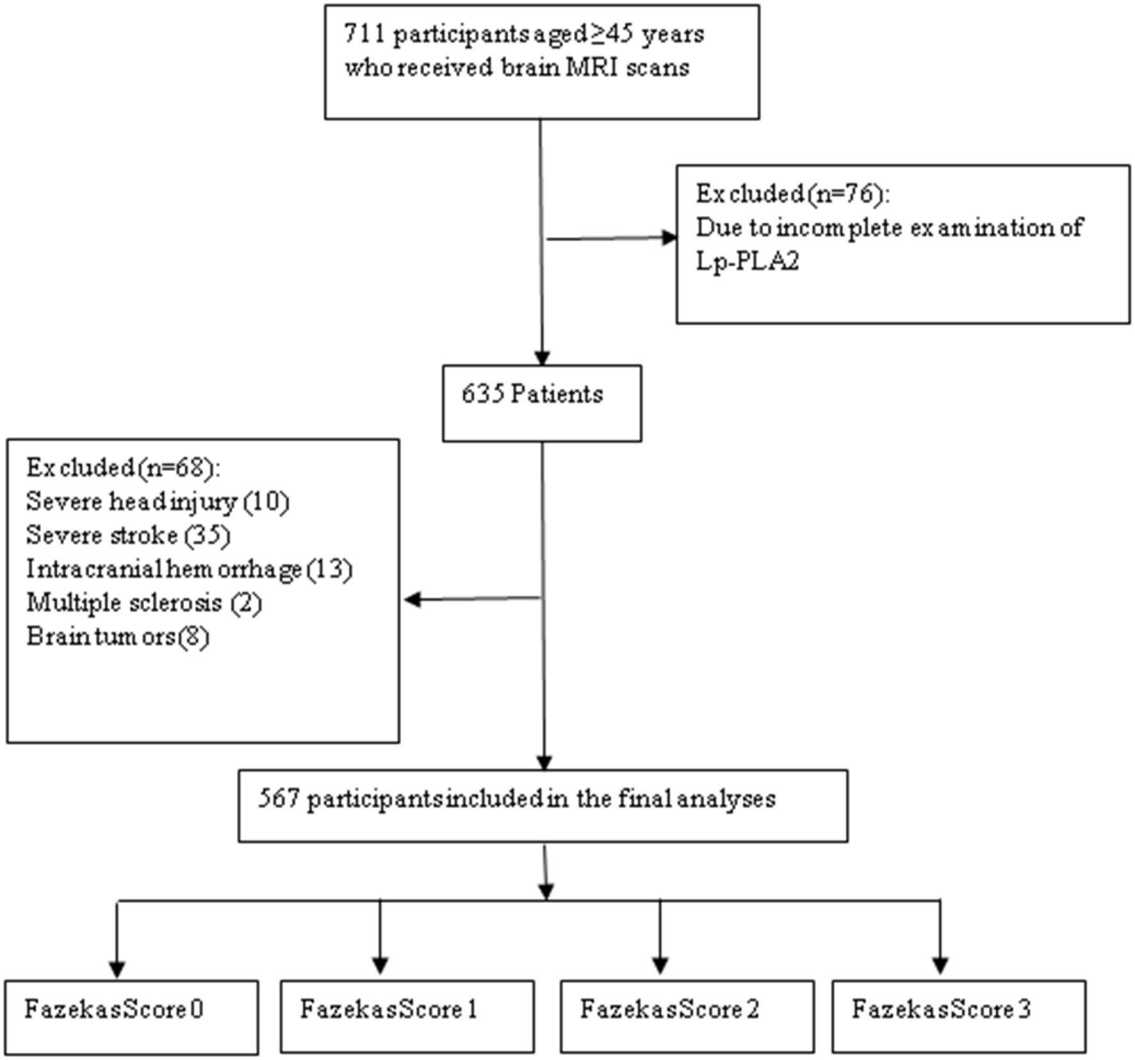
Study flowchart.

In this study, we included 711 cases of patients who received physical examinations in the Department of Neurology, the Affiliated Jiangning Hospital of Nanjing Medical University due to dizziness and headaches between January 2016 and July 2019. Finally, 567 subjects met the inclusion and exclusion criteria. The inclusion criteria were as follows: those aged ≥45years; those had received cerebral MRI. The exclusion criteria were as follows: those without Lp-PLA2 data, those with acute or chronic infectious diseases or rheumatic diseases, or those that may affect the evaluation of WMH, those with severe head injury, severe cerebral infarction, cerebral hemorrhage, multiple sclerosis, or brain malignancy. The study flowchart was shown in Figure 1. Each subject signed the informed consent. The study protocols were approved by the Ethical Committee of Affiliated Jiangning Hospital of Nanjing Medical University.

### Data Collection

The risk factors for cardiovascular diseases were collected from each subject, including clinical and demographic information. The information consisted of age, gender, hypertension, diabetes mellitus, coronary heart disease, atrial fibrillation, lacunar infarction, smoking, drinking alcohol, as well as formation of carotid artery plaque. In addition, laboratory tests were carried out to determine the fasting blood glucose (FBG), total cholesterol (TC), triglyceride (TG), low density lipoprotein (LDL), Hcy, Lp-PLA2, high-sensitive C-reactive protein (H-CRP), blood urea nitrogen (BUN), as well as creatinine (Cr). All the samples were collected from the venous blood of the subjects in a fasting state for 12 h. The serum Lp-PLA2 concentration was determined using the ELISA method, and was divided into normal range (<200 μg/L), moderate elevation (200–223 μg/L), and significant elevation (≥223 μg/L) according to the 2012 AACE' Guidelines for Management of Dyslipidemia and Prevention of Atherosclerosis (22). Immunoturbidimetry was used to determine the level of H-CRP, and the concentration of Hcy was determined using the enzymatic method. The diagnosis of carotid artery plaque was given based on the local intima media thickness (IMT) of >1.2 mm or a 1.5-fold or more to the peripheral IMT. The site for the quantification of IMT measurement was chosen in the area with a distance of about 1.5 cm to the carotid bifuracation (23).

### Cranial MRI Collection and WMH

**Figure 2:**
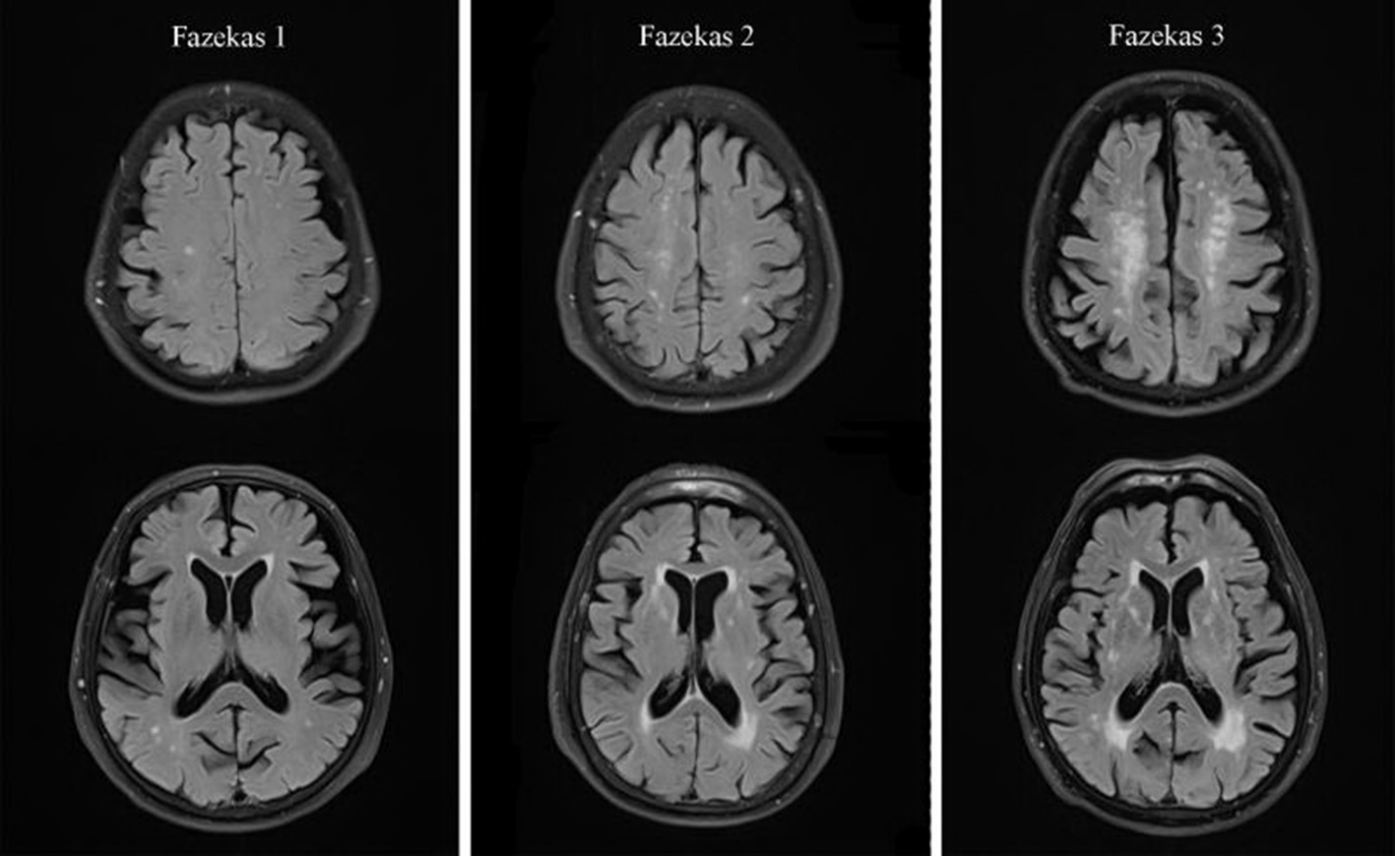
Representative T2-FLAIR images illustrating Fazekas score.

For the collection of cranial MRI, the MRI was performed using a 3.0T system, equipped with 8-channel coil arrays (Ingenia, Philips Medical System). MRI consisted of T1-weighted image, T2-weighted image, FLAIR image, and DWI findings. For the WMH, there was hyperintensity on T2-weighted images or FLAIR images in the lateral ventricle and subcortex region. WMH was confirmed in the presence of isointensity or hypointensity on T1-weighted signals (3). WHM was evaluated using the Fazekas scale (24). In the Fazekas scale, the lesions in the PVWMH and DSWMH were evaluated separately. Specifically, the PVWMH score standards were as follows: 0, loss; 1, calyptriform or pencil-like lamella; 2, smooth halo; 3, irregular hypertense signals near the ventricle extending to the deep white matter. The DSWMH score standards were as follows: 0, loss; 1, punctiform lesions; 2, initial fusion in the lesions; 3, massive fusion in the lesions (Figure 2). The Fazekas scale was evaluated by two experienced staff, specializing in radiation, who were blinded to the study, they reached a consensus through discussion to resolve their differences.

### Statistical Analysis

All the continuous data were presented as mean ± standard deviation, or the median (quartile). The categorical data were presented as the percentage. The continuous variables that were normally distributed were analyzed using the one-factor analysis of variance, while these that were not normally distributed were analyzed using the non-parametric test. The categorical variables were analyzed using the Chi square test. The independent risk factors for PVWMH and DSWMH were measured using the odds ratio (OR) or 95% confidential interval (95% CI), based on the ordinal regression analysis. SPSS 21.0 software was utilized for the statistical analysis. *P* < 0.05 was considered to be statistically significant.

## Results

### Characteristics of the Subjects

In total, 711 subjects were enrolled at first, among which 567 (male: 293; female: 274; median age: 65.51 ± 9.86 years) were finally included in this study. All the 567 subjects received Fazekas score evaluation. Finally, 468 cases (82.54%) were diagnosed with WMH, including 432 (76.19%) with PVWMH and 378 (66.66%) with DSWMH. Among these cases, 340 (59.96%) showed serum Lp-PLA2 of <200 μg/L, 26 (4.59%) showed serum Lp-PLA2 in a range of 200–223 μg/L, and the rest 201 (35.45%) showed serum Lp-PLA2 of ≥223 μg/L (Table 1).

**Table 1:** Baseline characteristics of the cohort.

**Clinical characteristics**	**Results**
Age, yr	65.51 ± 9.86
FBG, mmol/L	5.95 ± 2.12
TC, mmol/L	4.41 ± 1.05
TG, mmol/L	2.20 ± 9.62
LDL-C, mmol/L	2.41 ± 0.82
HDL-C, mmol/L	1.21 ± 0.34
H-CRP, mg/L	0.34 ± 0.08
BUN, mmol/L	5.50 ± 4.14
Cr, μmol/L	69.15 ± 21.23
Hcy, μmol/L	12.00 ± 7.80
Sex, men	51.68%
Hypertension, %	70.90%
Diabetes mellitus, %	23.28%
Coronary disease, %	9.70%
Atrial fibrillation, %	2.65%
Smoking, %	19.22%
Alcohol, %	12.00%
Carotid atherosclerosis, %	58.91%
WMH, %	82.54%
PVWMH, %	76.19%
DSWMH, %	66.66%
LI, %	49.74%
LP-PLA2 <200 ng/ml, %	59.96%

*WMH, white matter hyperintensity; PVWMH, periventricular WMH; DSWMH, deep subcortical WMH; LI, Lacunar infarction; FPG, fasting blood glucose; TC, total cholesterol; TG, triglyceride; LDL-C, low density lipoprotein cholesterol; HDL-C, high density lipoprotein cholesterol; BUN, blood urea nitrogen; Cr, creatinine; H-CRP, high C-reactive protein; Hcy, homocysteine; LP-PLA2, lipoprotein-associated phospholipase A2*.

### One-Factor Analysis for PVWMH and DSWMH

According to the PVWMH grouping, 135 subjects showed a Fazekas score of 0, 251 showed a score of 1, 107 showed a score of 2, and 74 showed a score of 3. There were statistical differences in the age, hypertension, diabetes mellitus, atrial fibrillation, lacunar infarction, Lp-PLA2 grade, creatinine, Hcy, and H-CRP (*P* < 0.05, Table 2). For the DSWMH grouping, 189 subjects showed a Fazekas score of 0, 244 showed a score of 1, 84 showed a score of 2, and 50 showed a score of 3. There were statistical differences in the age, hypertension, diabetes mellitus, lacunar infarction, Lp-PLA2 grade, TG, and Hcy (*P* < 0.05, Table 3).

**Table 2:** Clinical characteristics of subjects with PVWMH.

**Variable**	**Fazekas grade 0 (*n* = 135)**	**Fazekas grade 1 (*n* = 251)**	**Fazekas grade 2 (*n* = 107)**	**Fazekas grade 3 (*n* = 74)**	***P*-value**
Age, yr	59.83 ± 9.22	64.06 ± 8.98	70.44 ± 7.74	73.65 ± 8.01	<0.01
Men, n (%)	69(51.11)	120(47.81)	57(53.27)	47(63.51)	0.123
Hypertension, n (%)	71(52.59)	179(71.31)	86(80.37)	66(89.19)	<0.01
Diabetes mellitus, n (%)	17(12.59)	58(23.11)	34(31.78)	23(31.08)	<0.01
Coronary disease, *n* (%)	9(6.67)	21(8.37)	15(14.02)	7(9.46)	0.237
Atrial fibrillation, *n* (%)	0(0.00)	5(1.99)	6(5.61)	4(5.41)	0.019
LI, *n* (%)	19(14.07)	122(48.61)	74(69.16)	67(90.54)	<0.01
Smoking, *n* (%)	27(20.00)	44(17.53)	22(20.56)	16(21.62)	0.822
Alcohol, *n* (%)	20(14.81)	25(9.96)	15(14.02)	8(10.81)	0.473
LP-PLA2					
<200 ng/ml, *n* (%)	41(30.37)	57(22.71)	30(28.04)	12(16.22)	0.007
200–223 ng/ml *n* (%)	51(37.78)	109(43.43)	44(41.12)	22(29.73)	
>223 ng/ml, *n* (%)	43(31.85)	85(33.86)	33(30.84)	40(54.05)	
FPG, median (IQR)	5.37 (4.88–6.07)	5.44 (4.91–6.18)	5.39 (4.90–6.41)	5.31 (4.75–6.22)	0.850
TC, mean ± SD	4.57 ± 1.02	4.40 ± 1.04	4.26 ± 1.00	4.34 ± 1.19	0.305
TG, median (IQR)	1.43 (1.03–2.10)	1.58 (1.09–2.17)	1.34 (1.06–1.76)	1.53(1.02–2.24)	0.101
LDL-C, median (IQR)	2.46(1.96–2.98)	2.38(1.84–2.96)	2.25(1.73–2.79)	2.27(1.84–2.81)	0.465
HDL-C, median (IQR)	1.17(1.00–1.38)	1.18(0.99–1.35)	1.18(0.96–1.44)	1.10(0.97–1.39)	0.643
BUN, median (IQR)	5.02(4.02–5.90)	4.99(4.08–6.10)	5.10(4.11–6.40)	5.18(4.11–6.21)	0.526
Cr, median (IQR)	63.00(53.00–79.40)	65.00(55.00–77.00)	63.00(54.00–78.00)	70.50(62.98–86.13)	0.003
Hcy (IQR)	10.10(7.10–12.80)	10.40(7.93–13.20)	11.60(8.80–15.60)	12.65(9.80–15.58)	<0.01
H-CRP, median (IQR)	0.20(0.12–0.25)	0.30(0.24–0.35)	0.50(0.25–0.60)	0.60(0.34–0.70)	0.004

*WMH, white matter hyperintensity; PVWMH, periventricular WMH; DSWMH, deep subcortical WMH; LI, Lacunar infarction; FPG, fasting blood glucose; TC, total cholesterol; TG, triglyceride; LDL-C, low density lipoprotein cholesterol; HDL-C, high density lipoprotein cholesterol; BUN, blood urea nitrogen; Cr, creatinine; H-CRP, high C-reactive protein; Hcy, homocysteine; LP-PLA2, lipoprotein-associated phospholipase A2*.

**Table 3:** Clinical characteristics of subjects with DSWMH.

**Variable**	**Fazekas grade 0 (*n* = 189)**	**Fazekas grade 1 (*n* = 244)**	**Fazekas grade 2 (*n* = 84)**	**Fazekas grade 3 (*n* = 50)**	***P*-value**
Age, yr	60.40 ± 9.13	66.17 ± 8.93	69.30 ± 8.71	75.20 ± 7.42	<0.01
Men, *n* (%)	103(54.50)	115(47.13)	47(55.95)	28(55.95)	0.307
Hypertension, *n* (%)	112(59.26)	173(70.90)	73(86.90)	44(88.00)	<0.01
Diabetes mellitus, *n* (%)	34(17.99)	54(22.13)	30(35.71)	14(28.00)	0.012
Coronary disease, *n* (%)	12(6.35)	27(11.07)	9(10.71)	4(8.00)	0.365
Atrial fibrillation, *n* (%)	2(1.06)	7(2.87)	2(2.38)	4(8.00)	0.057
LI, *n* (%)	46(24.34)	130(53.28)	62(73.81)	44(88.00)	<0.01
Smoking, *n* (%)	44(23.28)	38(15.57)	18(21.43)	9(18.00)	0.221
Alcohol, *n* (%)	26(13.76)	25(10.25)	10(11.90)	7(14.00)	0.693
**LP-PLA2**					
<200 ng/ml, *n* (%)	59(31.22)	53(21.72)	25(29.76)	13(26.00)	0.038
200–223 ng/ml n (%)	71(37.56)	107(43.85)	27(32.14)	11(22.00)	
>223 ng/ml, *n* (%)	59(31.22)	84(34.43)	32(38.10)	26(52.00)	
FPG, median (IQR)	5.43 (4.93–6.19)	5.34 (4.80–6.18)	5.45 (4.89–6.47)	5.43 (5.08–6.17)	0.637
TC, mean ± SD	4.52 ±1.00	4.43 ± 1.03	4.08 ± 1.02	4.45 ± 1.27	0.059
TG, median (IQR)	1.43 (1.03–2.08)	1.56 (1.18–2.30)	1.34(1.00–1.82)	1.55(0.93–2.05)	0.038
LDL-C, median (IQR)	2.45(1.95–3.03)	2.36 (1.87–2.87)	2.16(1.53–2.64)	2.48(1.75–2.96)	0.052
HDL-C, median (IQR)	1.17 (0.97–1.40)	1.20 (1.00–1.38)	1.15(0.95–1.35)	1.17(0.97–1.48)	0.747
BUN, median (IQR)	5.01(4.10–5.90)	5.11(4.08–6.12)	4.77(3.93–6.22)	5.21(4.42–6.24)	0.623
Cr, median (IQR)	63.00(53.55–77.15)	65.00(56.00–77.93)	65.45(55.25–81.23)	70.50(60.35–88.03)	0.058
Hcy (IQR)	10.40(7.50–13.20)	10.40(7.93–14.28)	11.80(8.63–16.03)	12.60(9.98–15.10)	0.003
H-CRP, median (IQR)	0.20(0.15–0.25)	0.30 (0.24–0.35)	0.42(0.30–0.46)	0.45(0.28–0.48)	0.223

*WMH, white matter hyperintensity; PVWMH, periventricular WMH; DSWMH, deep subcortical WMH; LI, Lacunar infarction; FPG, fasting blood glucose; TC, total cholesterol; TG, triglyceride; LDL-C, low density lipoprotein cholesterol; HDL-C, high density lipoprotein cholesterol; BUN, blood urea nitrogen; Cr, creatinine; H-CRP, high C-reactive protein; Hcy, homocysteine; LP-PLA2, lipoprotein-associated phospholipase A2*.

### Independent Correlation Factors for PVWMH

To further evaluate the correlation factors for PVWMH and DSWMH, we established an ordinal regression model for the factors that may affect the PVWMH and DSWMH. Prior to the establishing of an ordinal regression model for PVWMH, we then performed the fitting test for the model (χ^2^ = 261.40, *P* < 0.001) and the linear hypothesis test (P=0.148), which indicated that the model showed good fitting. The ordinal regression analysis indicated that, after adjusting for age, hypertension, diabetes mellitus, atrial fibrillation, lacunar infarction, Cr, Hcy, and H-CRP, there was a lower risk of PVWMH in the patients with normal and moderately elevated serum Lp-PLA2 compared with those with significantly elevated serum Lp-PLA2 (normal Lp-PLA2: OR 0.258, 95% CI 0.119–0.520; moderate Lp-PLA2: OR 0.305, 95% CI 0.134–0.696) after adjusting for the confounding variables. In addition, PVWMH grade was positively correlated with the advanced age (OR 1.089, 95% CI 1.067–1.110), hypertension (OR 1.614, 95% CI 1.105–2.358), diabetes mellitus (OR = 1.579, 95% CI 1.078–2.314), as well as lacunar infarction (OR 5.155, 95% CI 3.540—7.508, Table 4).

**Table 4:** Ordinal regression model analysis of influencing factors of PVWMH classifications.

**Predictor**	**PVWMH Fazekas grade**					
	**Coefficient**	**Standard error**	**Z value**	***P*-value**	**OR**	**95%CI for OR**
Age	0.085	0.010	73.770	<0.01	1.089	1.067–1.110
Hypertension	0.479	0.193	6.143	0.013	1.614	1.105–2.358
Diabetes mellitus	0.457	0.195	5.502	0.019	1.579	1.078–2.314
Atrial fibrillation	0.214	0.503	0.181	0.671	1.239	0.462–3.320
LI	1.640	0.192	73.072	<0.01	5.155	3.540–7.508
LP-PLA2 <200^*^	−1.356	0.276	3.102	0.019	0.258	0.119–0.520
LP-PLA2 200–223^*^	−1.187	0.421	7.949	0.005	0.305	0.134–0.696
Cr	−0.003	0.004	0.408	0.523	0.997	0.989–1.006
Hcy	0.010	0.012	0.778	0.378	1.010	0.987–1.034
H-CRP	−0.002	0.007	0.107	0.743	0.998	0.984–1.011

LI, lacunar infarction; Cr, creatinine; H-CRP, high C-reactive protein; Hcy, homocysteine; Lp-PLA2, lipoprotein-associated phospholipase A2;

* *LP-PLA2 >232.00 ug/L served as control*.

### Independent Correlation Factors for DSWMH

After fitting test and linear hypothesis test, the ordinal regression analysis indicated a good fitting for the DSWMH (fitting test: χ^2^=198.546, *P* < 0.001; linear hypothesis test: *P* = 0.136). The ordinal regression analysis indicated that, after adjusting for age, hypertension, diabetes mellitus, lacunar infarction, Cr, TG, and Hcy, there was a positive correlation between DSWMH and advanced age (OR 1.081, 95% CI 1.061–1.102) and lacunar infarction (OR 3.823, 95% CI 2.662–5.490, Table 5).

**Table 5:** Ordinal regression model analysis of influencing factors of DSWMH classifications.

**Predictor**	**DSWMH Fazekas grade**					
	**Coefficient**	**Standard error**	***Z*-value**	***P*-value**	**OR**	**95%CI for OR**
Age	0.078	0.010	66.428	<0.01	1.081	1.061–1.102
Hypertension	0.348	0.194	3.221	0.073	1.419	0.969–2.071
Diabetes mellitus	0.272	0.192	1.996	0.158	1.313	0.900–1.912
LI	1.341	0.185	52.774	<0.01	3.823	2.662–5.490
LP-PLA2 <200^*^	−0.102	0.174	0.344	0.557	0.903	0.641–1.271
LP-PLA2 200–223^*^	−0.423	0.410	1.063	0.303	0.655	0.293–1.464
TG	0.027	0.025	1.109	0.292	1.027	0.977–1.080
Hcy	−0.000	0.011	0.002	0.969	1	0.979–1.020

LI, lacunar infarction; TG, triglyceride; Hcy, homocysteine; LP-PLA2, lipoprotein-associated phospholipase A2;

* *LP-PLA2 >232.00 ug/L served as control*.

## Discussion

Rare studies investigated the correlation between serum Lp-PLA2 and WMH. Previously, Lp-PLA2 was reported to associate with the WMH (25, 26). However, there are nearly no studies which have been conducted to illustrate the roles of serum Lp-PLA2 on the sites of WMH. In this study, we investigated the effects of serum Lp-PLA2 on PVWMH and DSWMH in the elder population. Ordinal regression analysis indicated that the patients with normal and moderately elevated serum Lp-PLA2 showed lower risks of developing PVWMH compared with those with significantly elevated serum Lp-PLA2. Our data revealed that statistical differences were merely observed between the patients with moderately and significantly elevated Lp-PLA2, which may be related to the fact that the sample size was not large. Moreover, no correlation was noticed between serum Lp-PLA2 and DSWMH. The serum Lp-PLA2 was independently correlated to the PVWMH rather than DSWMH. These findings indicated that Lp-PLA2 metabolic disorder played a crucial role in the pathogenesis of PVWMH.

WMH is featured by demyelination, loss of axon and gliosis (27). To date, there are still disputes on the pathogenesis of WMH. Inflammation was considered to be associated with the pathogenesis of WMH, however, in some circumstances, inflammation is a biological response to the infection and injury (28). Previously, inflammation was well-accepted to be closely related to the pathogenesis of WMH (14–16). In addition, there were regional variations in the effects of inflammation on the WMH. Inflammation was correlated with PVWMH rather than DSWMH (28), which was in line with our data. Compared with systemic inflammation biomarkers, there was a stronger correlation between vascular inflammation markers and WMH (28). In addition, uni-variate analysis indicated a correlation between H-CRP and PVWMH. After adjusting age, hypertension, diabetes mellitus, atrial fibrillation and lacunar infarction, creatinine, Hcy, and phosphatidase A2, there was no correlation between H-CRP and PVWMH. Similarly, there was no correlation between H-CRP and DSWMH. Nevertheless, systemic inflammation may contribute to the pathogenesis of WMH in the elder population in a recent prospective study involving a large sample size. This implied that systemic inflammation was correlated with the progression of CSVD, especially in the conditions with persistent inflammation (17). In future, further prospective cohort studies are required to illustrate the exact causes.

Lp-PLA2 is a type of enzyme involved in the hydrolysis of phosphoric oxide into oxidant fatty acids and lysophosphatidyl choline (23, 25). It is closely related to ischemic stroke (29) or vascular dementia (30). Besides, it contributes to the pathogenesis of atherosclerosis (31) and mediation of inflammation (29, 32). Moreover, it can mediate the vasculitis through regulating the metabolism of blood fat (33). To our best knowledge, few studies have been focused on the roles of Lp-PLA2 in the WMH. In a cross-sectional study involving 527 stroke-free subjects, Wright et al. indicated that Lp-PLA2 was associated with a greater burden of WMH independent of H-CRP. However, that study did not investigate the potential effects of Lp-PLA2 on the WMH in different regions (19). Our study indicated a correlation between elevation of serum Lp-PLA2 and PVWMH rather than DSWMH.

As is known to all, PVWMH usually occurs simultaneously with DSWMH. With the aging process, WMH usually happens in a single region of the white matter, which then progresses to another region, such as from the periventricular region to the deep subcortical region (20). Our data indicated that serum Lp-PLA2 showed various effects on PVWMH and DSWMH, however, we could not explain the exact mechanism and causes. To date, little is known about why inflammation induces various effects on PVWMH or DSWMH. In the previous studies (3, 21), PVWMH and DSWMH were considered to reflect various anatomical and histopathological features. In the anatomical view, there were anatomical differences between the arteriole in the peripheral ventricle and the deep subcortical region, however, the periventricular arteriola was superior to the deep subcortical arteriola in protecting the peripheral tissues from the vascular factors. In the histopathological views, DSWMH was likely to present more ischemic injuries, while PVWMH may involve more inflammatory metabolism (34–36). The inflammatory cascade reaction would trigger the endothelial dysfunction, which subsequently led to a slight dysfunction of the blood-brain barrier and WMH caused by tissue damages (27, 37). The calyptriform or smooth halo lesions in the peripheral ventricle were not originated from the ischemia. In fact, it was the subendymal gliosis and the demyelinated region beneath the ependyma lining. The deep subcortical punctiform signals, early fusion and fused white matter hypointensity indicated the gradual increase of the tissues with ischemic injuries, which was featured by slight perivascular lesions to massive axon loss, multiple small vacuoles and obvious arteriolosclerosis (34–36, 38). Therefore, these differences were tended to support the fact that serum Lp-PLA2 was more closely correlated to PVWMH rather than DSWMH.

Indeed, there are some limitations in this study. Firstly, we could not obtain a causal speculation based on these cross-sectional studies. Secondly, we only analyzed the circulating biomarkers at a certain time point. Therefore, prospective and longitudinal studies are required to illustrate the causal relationship between Lp-PLA2 and WMH. Thirdly, the Fazekas score was utilized for the grading of the WMH, without performing the quantification analysis. Fourth, more basic research is needed to further explore the pathogenesis of its differential effects.

## Conclusions

Serum Lp-PLA2 was closely related to the pathogenesis of PVWMH rather than DSWMH. Our data indicated that there might be different pathological mechanisms between PVWMH and DSWMH. In addition, Lp-PLA2 played an important role in the pathogenesis and progression of PVWMH.

## Data Availability Statement

The raw data supporting the conclusions of this article will be made available by the authors, without undue reservation.

## Ethics Statement

The studies involving human participants were reviewed and approved by Medical ethics committee of Affiliated Drum Tower Hospital of Nanjing University Medical School (2016YFC1300500). The patients/participants provided their written informed consent to participate in this study. Written informed consent was obtained from the individual(s) for the publication of any potentially identifiable images or data included in this article.

## Author Contributions

JJ and YG: design of the study. RZ, LW, XZ, WZ, QD, and XX: collection of data. YG and JJ: statistical analysis. JJ, YG, and XC: writing-review and editing of the drafts. All authors had full access to all the data in the study and take responsibility of the data and the accuracy of the data analysis.

## Conflict of Interest

The authors declare that the research was conducted in the absence of any commercial or financial relationships that could be construed as a potential conflict of interest.
